# Systemic immune effects of anesthetics and their intracellular targets in tumors

**DOI:** 10.3389/fmed.2022.810189

**Published:** 2022-07-28

**Authors:** Ting Luan, Yi Li, Lihui Sun, Siqi Xu, Haifeng Wang, Jiansong Wang, Chong Li

**Affiliations:** ^1^Department of Urology, The Second Affiliated Hospital of Kunming Medical University, Yunnan Institute of Urology, Kunming, China; ^2^Department of Anesthesiology, Peking University Third Hospital, Beijing, China; ^3^Institute of Biophysics, Chinese Academy of Sciences, Beijing, China; ^4^Zhongke Jianlan Medical Research Institute, Beijing, China

**Keywords:** volatile anesthetics, intravenous, opioids, local anesthetics, immune effect, tumor-targeting gene, tumor-associated signal pathway

## Abstract

According to the result released by the World Health Organization (WHO), non-communicable diseases have occupied four of the top 10 current causes for death in the world. Cancer is one of the significant factors that trigger complications and deaths; more than 80% cancer patients require surgical or palliative treatment. In this case, anesthetic treatment is indispensable. Since cancer is a heterogeneous disease, various types of interventions can activate oncogenes or mutate tumor suppressor genes. More and more researchers believe that anesthetics have a certain effect on the long-term recurrence and metastasis of tumors, but it is still controversial whether they promote or inhibit the progression of cancer. On this basis, a series of retrospective or prospective randomized clinical trials have been conducted, but it seems to be difficult to reach a conclusion within 5 years or longer. This article focuses on the effects of anesthetic drugs on immune function and cancer and reviews their latest targets on the tumor cells, in order to provide a theoretical basis for optimizing the selection of anesthetic drugs, exploring therapeutic targets, and improving the prognosis of cancer patients.

## Introduction

**FIGURE 1:**
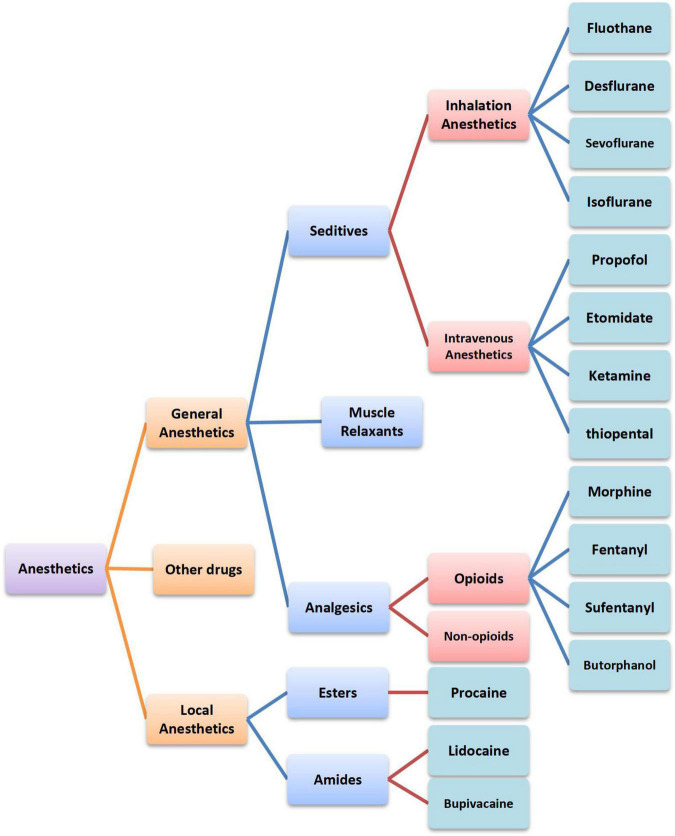
Classification of anesthetics commonly used in clinical practice.

Anesthetics are a diverse group of drugs that are used in the management of pains and are generally categorized into two classes according to their functions, namely, general anesthetics and local anesthetics (1). General anesthetics are either volatile liquids or agents that are administered intravenously to produce a state of unconsciousness so that invasive and surgical procedures can be carried out. Local anesthetics act on any part of the nervous system, causing both sensory and motor paralysis, which can be further divided into esters and amides according to the chemical structure (2). Despite the widespread use of anesthetics, the precise mechanisms of general anesthesia remain poorly understood. In addition, its influencing mechanism on tumors is unclear as well. In this article, we summarized the immune function of anesthetics commonly used in clinical practice (Figure 1). Furthermore, the latest acting sites of anesthetics on tumors were discussed. This review will provide a theoretical basis for optimizing the selection of anesthetics, exploring therapeutic targets, and improving prognosis and survival qualities of patients.

## Influence of anesthetics on immune function

Anti-tumor immunity includes innate and adaptive immunity. Anesthetic drugs can affect human body immune function. Normally, the immune system can recognize and remove mutated cells. However, some tumor cells have altered antigens, which help them escape from immune surveillance, resulting in their sustainable growth and metastases.

**FIGURE 2:**
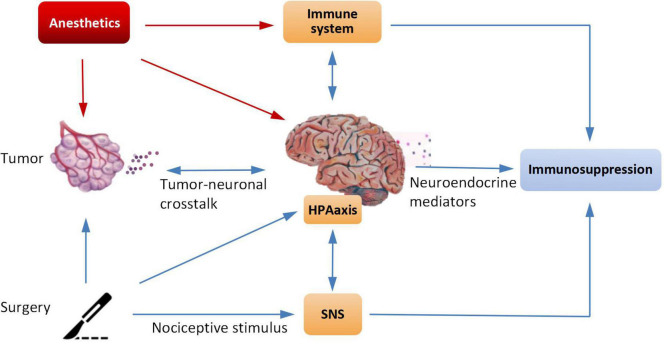
Anesthetics have effects on tumors, the nervous system, and the immune system.

With the increasing attention to cancer–nerve crosstalk, cancer biologists realize that the nervous system has an impact on tumors. Neural activity of the brain or spinal cord can directly promote the growth of cancer *in situ* or infiltration (3). Surgical resection induces noxious stimulation, and anesthetic factors induce immunosuppression, which can active the hypothalamic–pituitary–adrenal axis (HPA) and the sympathetic nervous system (SNS). The activation of these two systems suppress cell-mediated immunity and release immunosuppressive cytokines (4–6). The immune suppression caused by anesthetics plays an important role in the progression and metastasis of tumors (Figure 2).

### Influence of inhalation anesthetics on the immune system

Inhalation anesthetics with fluorinated ethers can act on the receptor of the central nervous system and generate anesthesia and sedation, as well as have direction actions on receptors located on the surface of immune cells, such as Ca/Mg ion channel protein, TLRs, integrin β2, and Ras1 protein (Rap 1), to promote degranulation of immune cells like NK cells and macrophages and consequently decrease their killing abilities and inhibit anti-tumor effects (5, 7). It has been revealed in a study that fluothane can affect the cytostatic activity of NK cells and increase the expression of hypoxia-inducible factor 1-alpha (HIF-1α) (8), decrease their secretion of IFN (interferon), and lower the killing abilities. Isoflurane can also decrease the cytostatic activity of NK cells, induce apoptosis of T and B lymphocytes, and reduce the Th1/Th2 ratio. Sevoflurane will contribute to the decrease in NK cells and increase the number of leukocytes and neutrophils at the same time (8, 9). Both sevoflurane and desflurane can have pre-treatment on neutrophils to inhibit the release of matrix metalloproteinase-9 (MMP-9) and then restrain the metastasis of colon cancer cells (10). *In vitro* experiments have shown that inhalation anesthetics can regulate immune cells to recognize antigens, recruit proinflammatory cells, and affect immune reactions mediated by cells (9). By dose-dependent effects, both sevoflurane and desflurane induce apoptosis of thymus T cells, and desflurane induces apoptosis of B lymphocytes by activating IP3 (inositol triphosphate) (11, 12), which might be the cause for immune suppression after operations.

### Influence of intravenous anesthetics on immune system function

Ketamine belongs to non-barbiturate intravenous anesthetics. Research studies suggested that ketamine and thiopental would have several implications on the immune system. Ketamine inhibits the cytostatic activity of NK cells, induces lymphocytic apoptosis *via* the mitochondrial pathway, and inhibits the maturation of dendritic cells. However, thiopental inhibits T-lymphocyte apoptosis by inducing heat shock proteins (HSPs) (4, 13). Propofol, which is different from other intravenous anesthetics, can increase the activity of NK cells, significantly inhibit the generation of COX-2 and PGE2, enhance the body protection against tumor immune reaction, and affect the tumor directly (14). Regarding adaptive immunity, disoprofol can increase the activity of cytotoxic T lymphocytes (CTL) and decrease proinflammatory cytokines, but it do not influence the Th1/Th2 ratio and can relieve the immune suppression caused by surgical traumas (15).

### Influence of opioid analgesics on immune system function

Immunocytes, such as neutrophils, macrophages, and T cells, can secrete endogenous opioid peptides (EOP), combine with the peripheric opioid receptor, and relieve inflammation and neuropathic pains (16). Meanwhile, immunologically competent cells also express opioid receptors and degrade the function of NK cells, macrophages, and subpopulation of B/T cells. For the mouse with the μ-opioid receptor (MOR) knocked out, using morphine does not affect the killing of NK cells (17, 18). Except for inhibiting the activity of NK cells, morphine can also decrease the expression of the toll-like receptor 4 (TLR4) of macrophages, inhibit the differentiation of T cells, and promote lymphocytic apoptosis (19, 20). It has been verified that opioid analgesics inhibit the proliferation of T lymphocytes, damage the killing function of T cells, and influence the production of antibodies (21, 22). Agonists of the MOR, such as fentanyl and sufentanyl, can inhibit cellular immunity of the body and the humoral immune system, as well as the activity of NK cells and macrophages and the production of antibodies (23). It was revealed in a colorectal surgery that sufentanyl lowers the rate of T-cell subsets, but remifentanil improved the IL-6 level and the secretion of hydrocortisone (24). Both fentanyl and sufentanyl with the clinical concentration can reinforce the suppression of the immune system by increasing the number of CD4, CD25, Foxp3, and T cells. In addition, fentanyl has a stronger inhibiting effect (25). Some research suggests that the expression of the κ-opioid receptor (KOR) exists in multiple types of blood cells, and the MOR activates the production of inhibiting antibodies and inhibits the evolution of T cells and the generation of inflammatory factors (26).

### Influence of local anesthetics on immune system function

Surgical traumas can activate the hypothalamic–pituitary–adrenal axis (HPA) of patients, which contributes to the change in neuroendocrine function and then inhibits immune functions. The use of local anesthetics can alleviate the stress reaction after operation and lower the inhibiting effect of stress on the immune system by blocking the signaling to nerves. Blocking peripheral nerves or intraspinal anesthesia can lower the conduction of stimulation caused by surgical injuries to the central nervous system and then reduce the immune suppression resulting from the activated HPA (27). Apart from the aforementioned indirect influences, local anesthetics can also directly have an effect on NK cells and T cells. Stress, pains, and opioids can inhibit the activity of NK cells (28–30), and the inhibiting or activating effect on NK cells brought by local anesthetics is related to their concentrations. Lidocaine, ropivacaine, and bupivacaine will inhibit NK cells when their concentrations are equivalent to infiltration anesthesia (31). However, with the intravenous concentration, lidocaine can enhance the activity of NK cells to resist tumor cancers by releasing lytic particles (32). Studies have shown that local anesthetics have analgesic and anti-inflammatory effects, as well as monitoring and protection of the immune system, which can improve the prognosis of cancer patients and improve disease-free survival (DFS) and overall survival (OS).

Based on the previous research, different anesthetics and methods can affect the immune function positively or negatively, and immune balance plays a role in tumor dissemination and recurrence. Depending on existing evidence, sevoflurane and isoflurane mostly play a negative role in anti-tumor immunity, but it is relatively vague for propofol. Local anesthetics can block neurotransmission from afferent nerves to the central nervous system, thus preventing surgical pains and reducing surgically induced neuroendocrine stress so that the HPA axis and SNS reactions are avoided. Toxicity that local anesthetics have on the cardiovascular system and the central nervous system, particular types of local anesthetics function, and their safe concentrations that effectively regulate the function of immune cells all need to be further studied.

## Study on influencing mechanism and acting site of inhalation anesthetics on tumors

The major review is that the influence of inhalation anesthetics on the OS of patients with cancers is stronger than that of intravenous anesthetics.

### Sevoflurane

Sevoflurane, a kind of inhalation anesthetics which is widely used in clinical practices, has many advantages, such as rapid recovery, strong controllability, and organ protection. The influence of evoflurane on tumors is currently being disputed.

Ru Li et al. made a comparative analysis between the respective use of sevoflurane and propofol in the mouse model of breast cancer and found that the mice with the adoption of sevoflurane had increased proinflammatory cytokines, which were related to tumor metastasis. On the first day after the operation, the volume of IL-6 and VEGF in the mouse serum with sevoflurane was higher than that in the group with propofol. Particularly, by increasing the expression of IL-6, sevoflurane activated the signaling pathway of IL-6/JAK/STAT3, induced the aggregation of CD11b bone marrow stromal cells in lung tissues, and promoted the metastasis of tumor cells to the lung. AZD1480, a JAK inhibitor, can be used to significantly reduce the number of metastasized tumors in the lung of the mice narcotized with sevoflurane and to reduce the level of p-STAT3 (33).

More studies put forward that sevoflurane promotes tumor, but some researchers hold the opposite view. Liang et al. revealed that sevoflurane could downregulate HIF-1α via the p38/MAPK signal channel and inhibit hypoxia-induced growth and metastasis of lung cancer cells (34). Similar research verified that sevoflurane inhibited the invasion and metastasis of colorectal cancer cells by regulating the ERK/MMP-9 pathway *via* miR-203 (35). Liang H et al. discovered that sevoflurane could lower the platelet aggregation rate (PAR) and inhibit the platelet activity by downregulating GPIIb/IIIa and CD62P, and then inhibit the invasion of lung cancer cells induced by platelet activity, which was not tested in the isoflurane group (36). The study in relation to breast cancer indicated that sevoflurane with low concentration significantly promoted the proliferation of primary cancer cells but remarkably inhibited metastatic cells, which indicated that sevoflurane had different effects on the proliferation of cancer cells at different stages (37).

Multiple reports show that sevoflurane affects the proliferation, apoptosis, migration, and invasion of cancer cells in relation to cervical cancer, optic glioma, stomach cancer, etc., which is currently not finally concluded and needs to particularly analyze its influence on the biological behavior of cancer cells of different types and statuses.

### Isoflurane

Isoflurane, a kind of inhalation anesthetics widely used in clinical practices, is thought to promote cancer progression by most of the research. However, it is believed from some of the results that isoflurane has inhibiting or no effects.

Research revealed that isoflurane could increase the expression of insulin-like growth factor (IGF), which stimulates the development of tumors. With isoflurane use, an increased expression of IGF-1 and IGF-1 RSKOV3 was found in ovarian tumor cells, which accelerated the cell cycle and proliferation (38, 39). Through the dependency mechanism of caveolin-1 (Cav-1), isoflurane resisted the apoptosis of colorectal cancer cells (40), increased the proliferation and invasion of squamous cell cancer of head and neck (SCCHN), inhibited apoptosis, and promoted cancer progression and metastasis (41). A report indicated that isoflurane upregulated the expression of HIF-1α in prostate cancer cells *via* the PI3K/AKT/mTOR signal pathway and contributed to the increased cell invasion and migration (42), but the use of propofol could reverse the activation of the signaling pathway involved, which indicated its application value in the operation of patients with tumors. The genotoxicity of inhalation anesthetics was closely related to the recurrence of tumor patients after operation (43).

Combined with statistical results of clinical samples, it is found that compared to total intravenous anesthesia, the use of isoflurane increases the risk of death in tumor patients, which indicates that propofol is more applicable to anesthesia in tumor excision surgeries. The anti-immune effect of isoflurane may not be applied to the population with low immunity but can be potentially used in the organ transplantation or patients with host immunity response stimulated by infection.

## Influence and acting sites of intravenous anesthetics on tumors

### Propofol

Propofol, an alkyl acid intravenous anesthetic with short-term effects which is widely used for intravenous injection in clinical practices, realizes sedative effects by enhancing the neurotransmission of central inhibition (the GABA pathway) and decreasing that of central excitation (the NMDA pathway) and is commonly used as an anesthetic in tumor resection operations. It is worthy of exploring whether propofol affects tumors when generating central sedation.

N-methyl-D-aspartate receptor (NMDAR), a subset of ionic glutamic acid receptor which is controlled by the membrane potential as well as by other neurotransmitters, plays an important role in the synaptic transmission and plasticity regulation in the central nervous system (44). By inhibiting glutamic acid release in the presynaptic element, propofol with clinical concentration induces the allosteric regulation of various sub-units of NMDAR, conducts negative controls, and produces sedative effects (45). After NMDAR is activated, the ion permeation of Ca2 + and K + is increased, which causes a large number of Ca2 + to flood into the cell; thus, the associated transcription, translation, and post-translational modification are increased as well. At present, the related research is concentrated in the cognitive deletion caused by abnormal activities of NMDAR, such as research in relation to epilepsy, Alzheimer’s disease (AD), Parkinson’s disease (PD), and anti-NMDAR encephalitis (46); however, rare studies involve the relation between NMDAR and tumors as well as the mechanism of action. Based on the testing and screening on lung cancer, colon cancer, breast cancer, prostate cancer, stomach cancer, liver cancer, esophageal cancer, and cervical cancer, some researchers found that NMDAR was positively expressed in tumor tissues, which was significantly different from normal paracancer tissues (*P* < 0.05) (47), but the research did not further clarify the effect that NMDAR had on the biological behavior of tumors. North et al. reported the positive expression of NMDAR in neuroblastoma and found that the use of glutamic acid and NMDAR agonist could trigger the change in the ion channel which had toxic injuries on neuroblastoma cells (48). Sub-types of NMDAR were also tested in the stomach cancer cell line, and with the use of AP-5, a retardant of NMDAR, proliferation of stomach cancer cells was effectively inhibited (49). Similar reports of research related to breast cancer, particularly, NMDAR, was expressed negatively in normal breast tissues but positively in the breast cancer cell line and patients’ breast cancer tissues. In addition, MK-801, another NMDAR retardant, could significantly inhibit the proliferation of breast cancer cells (50). A recent study revealed that the expression of NMDAR in colorectal cancer was significantly different from that in normal tissues, and as an important marker of endothelial cells, NMDAR promoted tumor angiogenesis and then facilitated tumor progression (51).

GABAR, the receptor of γ-aminobutyric acid, is the another crucial acting site of propofol. As the most significant inhibitory neurotransmitter in the central nervous system of mammals, by combining with GABAR, the receptor can lead to changes in ion permeation in the cell membrane. There are three pharmacological subtypes of GABAR, particularly, GABAR(A), GABAR(B), and GABAR(C). By combining with receptors of different subtypes, GABA will produce different regulatory effects *via* the specific signal transduction pathway. Recent research indicates that except for the existence of GABA and GABAR (the receptor of GABA) in the central nervous system, with their expressions in some tumor tissues, they regulate tumor proliferation, invasion, and metastasis and engage in the development and progression of tumors *via* specific signal transduction pathways. The research suggests GABA is significantly expressed in tumor tissues such as neuroglioma, prostate cancer, and colorectal cancer, but the expression of GABAR is significantly increased in liver cancer and breast cancer (52–54), which indicates that the high expression of GABA and its receptor in tumor tissues is potentially related to tumor development. The expression of GABAR is increased in the mouse with liver cancer which is induced by N-nitrosodiethylamine. Mitosis and DNA synthesis of liver cancer cells can be promoted with the adoption of the GABAR agonist. Inamoto et al. (55) studied the regulatory effect that GABA had on activating the MAPK signal pathway in Caki-2 (human renal clear cell carcinoma cell line) and found that GABA activated receptor B to promote phosphorylation of the MAPK family, which included ERKl/2, cJNK, and P38, and then improved the invasion ability of renal cell carcinoma. However, another research results showed that (56–58) the adoption of the GABAR agonist can inhibit the proliferation of tumor cells. By increasing the activity of GABAR, muscimol effectively lowers the expression of alpha fetoprotein (AFP) in HepG2 (human liver cancer cell line), as well as cell proliferation, and a similar result is also obtained in research on colorectal cancer and stomach cancer, which reveals that the intervention of the GABAR pathway is potentially an effective therapeutic method for gastroenteric tumors.

Due to differences of the structure and function between tumor cells and neuronal cells, the GABAR pathway in neuronal cells is different from that in tumor cells. It needs to be further explored by researchers with regard to whether the expression of GABA and GABAR in tumor tissues is a tumor promoter or a protective response of the body, what kind of receptor pathway is adopted to play the regulatory role, and what is the signal transduction pathway.

### Etomidate

Etomidate, a kind of non-barbiturate intravenous anesthetics and a derivant of imidazole, produces analgesic effects by activating GABAR (59, 60). It is especially suitable for anesthesia induction on critical patients and can also be applied to undergoing little operations due to its slight influences on hemodynamics, short action time, and rapid onset (61). Currently, there is few research on the influence of etomidate on tumors.

A research indicated that with the adoption of etomidate in resection operation of lung adenocarcinoma, the quantity of CD8 + T cells in patients’ blood after the operation was lower than in that in the propofol group but higher than that in the propofol group 24 hours after the operation, which illustrated that etomidate had smaller effects on the immune system of patients with lung adenocarcinoma (62), and its adoption could improve immune suppression of the body in the perioperative period. In another research on lung cancer, after A549 (human lung adenocarcinoma cell line) treated with etomidate, the activation of MMP2 was inhibited, and expressions of PKC, MMP7, MMP1, MMP9, and p-p-38 were significantly downregulated, but those of RAS, PI3K, and P-ERK (phosphorylation extracellular signal-regulated kinase) were upregulated; therefore, the migration and invasion of A549 were inhibited (63). Etomidate contributed to the loss of mitochondrial membrane potential (MMP) in N2a cells; produced reactive oxygen species (ROS); promoted the generation of apoptin such as PARP, caspase-9, and procaspase3; and facilitated the apoptosis of neuroblastoma Neuro-2a cells (N2a) (64). Limited research reveals the inhibiting effect that etomidate has on tumors, which still needs to be verified by basic experiments and clinical trials with large-scale samples.

As one of the most commonly used intravenous anesthetics, only a few studies have shown that propofol can promote the proliferation of some cancer cells, and clinical data show that propofol has no great influence on long-term prognosis. But most cytological and animal studies suggest that propofol seems to have a tumor-suppressive effect by decreasing cancer cell migration, invasion, proliferation, and angiogenesis and by inducing apoptosis, as demonstrated in many experimental studies. At present, few research studies are found on the effects of etomidate on tumors. It is still necessary to further reveal the regulatory effect and molecular mechanism of propofol and etomidate in different types of cancers. Clinical studies are also needed to confirm their effects and provide a reference for the rational selection of propofol or etomidate.

## Influence and acting sites of opioid analgesics on tumors

The opioid receptor, a kind of G-protein-coupled receptors (GPCRs) with seven transmembrane domains (TMDs), mainly exists in the central nervous system with four subtypes (μ, κ, δ, and ORL1), namely, MOR, KOR, DOR, and ORL1 receptors. With approximately 60% of amino acids are structurally identical, these receptors of different types could have analgesic, sedative, and other effects after being activated (65, 66). Emerging research has suggested that the expression of opioid receptors is detected in tumor cells such as lung cancer, prostate cancer, breast cancer, and liver cancer (67–69). The opioid drug indicates a kind of drug that is naturally created or partially synthesized and produces reactions by combining with opioid receptors and is the most effective analgesics available. Due to its strong analgesic effects, opioid drugs are widely applied to the abirritation in the perioperative period and treatments of postoperaticve pains and chronic cancer pains. The result of recent research indicates that the effect that opioid drugs have on patients with tumors is greatly disputed.

### Morphine

Morphine is the agonist of MOR, KOR, and DOR, which has gradually decreased effects on subtypes of the aforementioned three receptors. It is currently believed that the effect that morphine has on tumor development is contradictory, and both its promoting and inhibiting effects are reported. Based on a cytological study, it was found that morphine could activate the MOR in lung cancer cells, induce phosphorylation of the epidermal growth factor receptor (EGFR), lead to activation of the lower MAPK/ERK/Akt pathway, and promote cell proliferation and invasion. Increasing expressions of the EGFR and MOR in lung cancer could promote growth of tumor cells, trigger angiogenesis mediated by the vascular endothelial growth factor (VEGF), increase vascular permeability, accelerate tumor progression, and increase the risk of micrometastasis (70).

However, there is also evidence that morphine can inhibit tumor progression. It was revealed in an *in vivo* and *in vitro* research on nude mice that a 24-hour incubation of 10 μM morphine could significantly increase the apoptosis rate of Hep3B/HepG2 (71), while 5 μM or 10 μM morphine significantly decreased proliferation and invasion of liver cancer cells and lowered the occurrence rate of pulmonary metastasis. By upregulating OGFR and downregulating MOR, uPA, and MMP-9, morphine inhibited the biological characteristics of tumors. In addition, morphine with the clinical concentration could significantly inhibit tumor progression, which is thought to be a safe and effective pain treatment for patients with liver cancer. Another data also supported the fact that morphine could affect the opioid growth factor receptor (OGFR) and finally inhibit proliferation of lung cancer cells (72). OGFR, the ζ-opioid receptor, does not relieve pain, which differs from the common μ-opioid receptor, κ-opioid receptor, and δ-opioid receptor. The expression of the OGFR in lung cancer tissues is significantly higher than in para-carcinoma tissues, and morphine plays a role in the OGFR and inhibits cell proliferation. Zagon et al. tested 31 cases of human tumor cell lines and found that 90% of the tumor cells had a high expression of the OGFR, and 42% of cell proliferation was decreased after OGF was added into the culture dish, but 44% was accelerated after naltrexone (opioid receptor antagonist) was added (73), which indicates that application of morphine in the treatment of pains for patients with tumors is safe. Based on research on colorectal tumors, it was found that morphine could significantly inhibit lipopolysaccharide (LPS) and decrease the expression of endothelial cell adhesion molecules and therefore inhibit the tumor progression induced by LPS and prevent tumor growth and metastasis (74).

### Fentanyl

Fentanyl, which belongs to short-acting opioid analgesics, is a strong MOR agonist with powerful analgesic effects, with no influences on the respiratory system within the scope of safe dose, fairly stable hemodynamics, short acting time, and fast metabolism. Increasing research has testified that the expression of the MOR exists not only in the central nervous system but also in many tumor cells.

Compared to normal lung tissues, the expression of the MOR in tumor tissues of patients with non-small-cell lung cancer (NSCLC) was 5∼10 times increased, which was consistent with that in the NSCLC cell line, which indicates that high expression of the MOR may have regulated a series of biological characteristics including cell proliferation and differentiation (75, 76). A retrospective analysis of about 113 cases of patients with prostate cancer shown that the increased expression of the MOR predicted a poor survival rate of patients (77). It was speculated by some researchers that the MOR could affect various aspects such as angiogenesis and immune regulation by activating signal pathways of PI3K, Akt, and mTOR, and promote tumor recurrence and metastasis (74, 77–79). Except for the central nervous system, opioid receptors also exist in multiple kinds of stem cells of the body, such as neural stem cell, embryonic stem cell (ES cell), bone marrow mesenchymal stem cell (BMMSC), and epidermal stem cell, which play an important role in regulating proliferation and differentiation of nerve cells, improving ventricular remodeling (VR) and healing wounds. Our previous research indicated that acting as one of the biomarkers of liver cancer stem cells (LCSCs), the high expression of MOR promoted the self-renewal of LCSCs (69).

Although most of the research results revealed that the activated MOR could promote tumor progression, for patients with advanced tumors, the effect of the MOR agonist on the opioid receptor in the central nervous system could effectively relieve their pains, reduce inflammatory reactions caused by tumors, inhibit angiogenesis, and alleviate tumor recurrence and metastasis (78). Research suggested that fentanyl could directly work on human pancreatic cancer cells to inhibit their activity and lower the speed of tumor growth (79). It was also revealed in similar research results that fentanyl could decrease miR-182 and MMP-9 generated by β-catenin to inhibit the growth and invasion of tumor cells for colorectal cancer (80). The direct biological effects that opioid drugs have on tumors are closely related to the drug type and administration concentration and method.

### Butorphanol tartrate

The major metabolite of butorphanol tartrate activates KOR and has double actions on the MOR particularly, as the activator and the inhibitor. It majorly plays its pharmacological role indirectly by combining with the receptor in the central nervous system.

Currently, there are few research studies on KOR and tumors, which is available in relation to studies on liver cancer, bone cancer, prostate cancer, and melanoma. Most of the research studies were inclined to support that the KOR had anti-tumor effects and patients with low expression of KOR had poor survival rates (81). A study on bone cancer believed that the KOR agonist could effectively relieve pains but would not promote tumor progression synchronously (82). The application of U50 and 488H (KOR agonists) combined with the targeted therapeutic drug gefitinib could significantly inhibit the growth of NSCLC cells by activating the phosphorylated glycogen synthase kinase 3β pathway (83). KOR had the effect of inhibiting angiogenesis, and it could relieve pains and inhibit tumor progression by promoting apoptosis (84–86), which indicates that KOP is potentially a new target to treat tumors. A research on human epithelial cancer cells thought that the KOR promoted apoptosis of tumor cells *via* the PKC or Bcl-2 pathway (85, 86). However, there were also opposite views which believed that the expression of the KOR was in positive correlation to lymph node metastasis of esophageal cancer, and the KOR was an independent prognostic factor, which indicated poor prognosis (87). Currently, there is no convincing evidence to testify that through kinds of the pathway the KOR affects in tumors and influences patients’ survival; therefore, more experimental data associated are wanted to support the view.

Evidence on the association of opioids with tumor recurrence and prognosis in cancer patients is still lacking. Current evidence mainly comes from cell and animal experiments, and some retrospective studies and small randomized controlled studies have emerged. However, there is still a lack of evidence from large-scale randomized controlled clinical studies. Due to mixed factors which were related to the type, administration method, and use time of opioid drugs clinically, it was really difficult to determine the “direct effects” that opioid receptors had on tumor development and progression by clinical studies alone, and research on the associated mechanism is an urgent need (88, 89). Overemphasizing the potential role of opioids in promoting tumor recurrence and metastasis and limiting their application in the perioperative period may harm the interests of patients. Opioids remain an important component of perioperative analgesia.

## Influence and acting sites of local anesthetics on tumors

Local anesthetics are a kind of drugs applied to block the occurrence and conduction of nerve impulses to make short-term and reversible analgesia occurred in innervation areas. Based on different chemical structures, local anesthetics can be divided into esters and amides. Common esters include procaine, tetracaine, and cocaine, and amides often include lidocaine, bupivacaine, and ropivacaine.

In recent years, it was found in the research that local anesthetics could have effects on proliferation, invasion, and metastasis of multiple types of tumor cells with different mechanisms of action and, in addition, their different administration methods would also influence recurrence, metastasis, and prognosis after the tumor operation (90). Through different administration methods, such as local infiltration and intravenous, intraspinal, and regional block, local anesthetics provide the personalized treatment plan for tumor patients in the perioperative period. The discussion on the mechanism of action in tumor metastasis and recurrence can provide new strategies and basis for the appropriate use of local anesthetics in tumor patients in the perioperative period.

### Esters

It was found in the research that as a kind of ester local anesthetics, procaine with 10 nM concentration could inhibit A549 proliferation. In an animal experiment, 3-week administration of procaine on the mice with lung cancer (50 mg/kg/day) could significantly reduce the expression of the EGFR and the tumor volume (91). By increasing demethylation, procaine inhibited the growth of breast cancer cells, made 5-methylated DNA 40% be downregulated in MCF-7 (breast cancer cell line), and made high-methylated tumor suppressor genes be demethylated, which indicated that procaine had high application value in patients with targeted drug resistance (92). The *in vitro* experiment illustrated that procaine could effectively inhibit the growth of HLE, HuH7 and HuH6 (human liver cancer cells) and had demethylation effects, which was potentially a candidate drug for liver cell cancer (93). The associated research also revealed that procaine promoted proliferation arrest and apoptosis *via* the regulation of DNA methylation in stomach cancer, which indicated that procaine had specific anti-tumor potentials (94). The application of procaine in research on nasopharyngeal cancer could significantly inhibit the proliferation of CNE-2Z and make cells be arrested before the G1 phase, which was presented in relations of time dependency and dose effect (95).

### Amides

#### Lidocaine

The effects that different types of amide local anesthetics had on tumors differed from others, particularly lidocaine and ropivacaine increased demethylation of breast cancer cells, but bupivacaine did not (96). Lidocaine, which was presented in time and dose dependency, could increase the demethylation of breast cancer cells, and the methylation-inhibiting effect of DAC (5-azacytidine), which is a kind of DNMTs (DNA methyltransferases), could decrease the CpG (CpG island) methylation of tumor suppressor genes—*RAR*β and *RASSF1A*, and increase the sensitivity of cisplatin to breast cancer cells (97, 98). The infiltration anesthesia concentration of lidocaine commonly used in clinical practices is 0.5%∼2% (19mM∼74mM), and the regional concentration after being spread in the tissue is about 0.4mM. The research suggested that lidocaine with 0.4mM concentration could inhibit the EGFR by combining with EGF to generate phosphorylation and lower proliferation of corneal epithelial cells (99), as well as inhibit the growth of CAL27 (tongue cancer cell line) by decreasing EGFR phosphorylation (100).

miRNA can be involved in regulating the proliferation, differentiation, migration, angiogenesis, and apoptosis of tumor cells. As an important member of the miR-520 family, the decreased expression of miR-520a-3p is in connection with the development and progression of multiple tumors such as colorectal cancer and NSCLC. Lidocaine with 0.5 mM concentration could decrease the expression of EGFR by upregulating the level of colorectal cancer cells and miR-520a-3p (retinoblastoma cell line) and inhibit the proliferation of tumor cells (101, 102). However, in lung cancer, lidocaine with a higher concentration (8 mM) was required to promote miR-539, inhibit the expression of EGFR as well as its ERK (extracellular regulated protein kinases), and the PI3K/Akt signal pathway to play its role in inhibiting the proliferation, migration, and invasion of tumor cells (103, 104).

#### Bupivacaine

Bupivacaine is a kind of long-term amide local anesthetic, which has strong combination with plasma proteins. The toxicity of bupivacaine is four times greater than lidocaine, its cardiotoxicity should be noticed especially, and the rate between circulatory collapse and convulsions (CC/CNS) induced by is low.

The degree of apoptosis induced by lidocaine and bupivacaine in MCF-7 (breast cancer cell line) was significantly higher than that in MCF-10A (mammary epithelial cell line), both of which could activate caspase 7, caspase 8, and caspase 9 in MCF-7 and promote the lysed nucleosome and thus induced the apoptosis of MCF-7 *via* intrinsic and extrinsic pathways of mitochondria (105). Chang et al. (106) proved in their studies that with the concentration-dependent method, lidocaine and bupivacaine affected the cytotoxicity of thyroid cancer cells and inhibited the MAPK pathway to lower the survival and colony formation of 8505C and K1 (thyroid cancer cell lines) and induce the apoptosis of cancer cells (106). Lidocaine and bupivacaine could lead to the division of mitochondrial membrane protein and release of cytochrome C, activate caspase 3 and caspase 7 synchronously, and contribute to the break of poly ADP-ribose polymerase (PARP) and Bax/Bcl-2 with a higher rate. They could weaken the activity of ERK1/2 (extracellular signal-regulated protein kinases) and promote the activation of caspase 3 and break of PARP through inhibiting mitogen-activated protein kinase (MAPK) and c-Jun N-terminal kinase (JNK).

Current literature has pointed out that the intravenous infusion of lidocaine provides an antiproliferative tumor suppressive effect. Many molecular pathways/mechanisms are described. Local anesthetics could directly act on tumor cells and induce apoptosis of tumor cells through intrinsic and extrinsic pathways (107). Lidocaine used in the perioperative period benefited postoperative analgesia and inflammatory reaction control, had protective effects on immune surveillance of the innate immune system, improved the disease-free survival rate and the overall survival rate *via* migration of anti-tumor cells, and improved the prognosis of patients with tumors, which was possibly an ideal adjuvant drug in the treatment of cancers. However, it should be further verified by clinical research on how it can be clinically used.

## Conclusion

Cancer is a disease with high heterogeneity. Various factors contribute to the activation of oncogenes or mutation of tumor suppressor genes, which results in great differences between the therapeutic effectiveness of cancers.

Surgical stress and immunosuppression are unevadable topics. With the activation of the HPA axis and SNS, neuroendocrine regulators are increased, which promotes the metastasis of residual or circulating tumor cells to regional lymph nodes and distant sites. We can reduce the noxious stimulus as much as possible by prioritizing the selection of local anesthetics. In the perioperative period, anesthetics and analgesics are inevitable environmental exposure factors for patients with tumors. As shown in Tables 1, 2, increasing evidence has indicated that anesthetics can directly act on the immune function and relevant sites in tumor cells to activate a series of signal transduction pathways and affect tumor proliferation, invasion, migration, etc. The aforementioned findings suggest that different anesthetics have different or even opposite effects on anti-tumor results. The mechanisms are mainly related to cell cycle regulatory pathways and immune pathways. Meanwhile, animal models greatly benefit research on the mechanism of action that anesthetics have in tumor metastasis, which will provide reference for clinical medication as well as a basis for overcoming side effects of anesthetics. These findings will provide potential research and application directions for rational drug use in different surgeries and patients.

**TABLE 1:** Influence of various anesthetics on immune function.

Agent	Neutrophil	NK cell	Macrophage	T cell	B cell	Refs.
**Volatile anesthetics**						
Sevoflurane	Inhibit N recruitment via inhibiting LFA-1; down-regulate PRRs, reduce inflammatory reaction, and inhibit release of MMP-9 from N	Reduce the cytotoxicity of NK	Preserve the function of macrophage *in vitro* experiment;	induce apoptosis of thymic T cells; Low-flow sevoflurane alleviates T cell suppression	reduce the number of peripheral blood lymphocytes and spleen B cells in mice	(9–11, 108–110)
Isoflurane	Inhibits the adhesion of neutrophils to human endothelial cells	Suppress activity of NK cells	Inhibit macrophage recruitment via inhibiting Mac-1;	induce apoptosis of thymic T cells, induce apoptosis of T lymphocyte, lower the rate of Th1/Th2;	induce apoptosis of B lymphocyte via activating IP3	(11, 12, 108, 111, 112)
**Intravenous anesthetics**						
Propofol	Inhibit the immune function of neutrophils;	Increase the activity of NK cells, reduce proinflammatory cytokines; no effect on NK;	Recruit macrophages to liver cancer cells;	increase the activity of CTL, but not affect the rate of Th1/Th2		(14, 15, 108, 113, 114)
Etomidate	reduce inflammatory response	No effect on NK cytotoxicity;		Maintain the level of CD4 + and CD8 + T cells, reduce immune suppression		(62)
**Opioids**						
Morphine	Combine with the G-protein coupled μ receptor on the surface of immune cells, produce immune suppression;	Suppress activity of NK cells	downregulate the expression of TLR4	block signal transduction of TCR, and inhibit its function	Attenuate B cell producing anti-tumor antibodies;	(19, 20, 115–117)
Fentany		Inhibit the activity of NK cells;	Inhibit macrophages	increase the number of CD4 + CD25 + Foxp3 + T to inhibit the cell immune system	Inhibit humoral immune system	(23, 25)
Butorphanol tartrate	Inhibit generation of inflammatory factors			inhibit T-cell evolution	Activated KOR inhibit generation of antibodies,	(26)
**Local anesthetics**						
Lidocaine	Lidocaine reduces neutrophil adhesion, producing anti-inflammatory effects	Increase activity of NK cells via releasing lytic granules	Decreased inflammatory cytokine expression in dendritic cells and macrophages	inhibition of Th1 differentiation		(32, 118)
Bupivacaine		Inhibit NK cells with the concentration of infiltration anesthesia				(31)

**TABLE 2:** Influence of tumor-targeting genes and signal transduction pathways in tumor cells.

Agent	Tumor-targeting gene	Tumor-associated signal pathway	Refs.
**Volatile anesthetics**			
Sevoflurane	Promote lung metastasis of breast cancer via miR-203	Promote IL-6/JAK/STAT3 pathway, down-regulate HIF-1α via p38/MAPK signal pathway; regulate ERK/MMP-9 pathwayto inhibit invasion and migration of colorectal cancer cells	(33, 35)
Isoflurane	Promote expression of IGF; up-regulate expression of HIF-1α in prostate cancer cells, contribute to increase of invasion and migration of tumor cells	PI3K/AKT/mTOR pathway	(38, 42)
**Intravenous anesthetics**			
Propofol	Promote over-expression of Nrf2, and promote proliferation of esophagus cancer cells; inhibit proliferation and metastasis of esophageal esophagus cancer cells; inhibit activity of HIF-1 and gene expression; phosphorylation of ERKl/2, cJNK and P38; up-regulate miR-142-3p via secreting microvesicles, and inhibit metastasis of liver cancer cells;	Wnt/β-catenin signal pathway, NMDAR-CAMKII-ERK pathway, ERK1/2-dependent PUMA signal pathway ERK/VEGF/MMP-9 Inhibit PI3K/AKT/mTOR/HIF-1α MAPKs signal pathway activity	(42, 55, 114, 119–124)
Etomidate	miR-211-5p/ROBO1 inhibit proliferation, invasion and migration of stomach cancer cells; down-regulate MMP2, MMP7, MMP1, P-P-38, etc. to inhibit migration and invasion of A549 (lung cancer cell line); promote generation of apoptins PARP, caspase-9 and procaspase3, and promote apoptosis of N2a (neuroblastoma Neuro-2a cells)	Activate RAS/PI3K/P-ERK signal pathway	(63, 64, 125)
**Opioids**			
Morphine	Act on OGFR to inhibit proliferation of lung cancer cells; activate MOR; induce phosphorylation of EGFR; up-regulate OGFR and down-regulate MOR, uPA and MMP-9 to inhibit tumors	Promote activation of MAPK/ErK Akt to facilitate proliferation and invasion	(70–72)
Fentany	Act on MOR in tumor cells; reduce miR-182 and MMP-9 generated by β-catenin, inhibit growth and invasion of tumor cells in colorectal cancer	MOR promote tumor recurrence and metastasis via activating PI3K, Akt and mTOR signal pathways	(75, 80, 116, 126, 127)
Butorphanol tartrate	Act on KOR in tumor cells	Inhibit tumors via activating pGSK-3β pathway; inhibit tumor angiogenesis via interfering VEGF signal pathway; promote apoptosis of tumor cells via PKC or Bcl-2 pathway	(83–86)
**Local anesthetics**			
Lidocaine	Up-regulate miR-520a-3p, reduce expression of EGFR, and inhibit proliferation of tumor cells	Inhibit ERK/PI3K/Akt signal pathway, inhibit proliferation, migration and invasion of tumor cells	(101–103)
Bupivacaine	Up-regulate RASSFFIA mRNA, inhibit proliferation of CNE-2Z (nasopharyngeal cancer cell line); inhibit activity of DNMT1/DNMT3A	Inhibit MAPK pathway, induce apoptosis of cancer cells; decrease activity of ERK1/2	(95, 106)

Furthermore, it is necessary for scientists of clinical medicine to conduct a series of retrospective or perspective and stochastic clinical trials to explore the effect that anesthetics have on long-term recurrence and metastasis of tumors, such as to provide a theoretical basis for optimizing the selection of anesthetics, exploring therapeutic targets, and improving the life quality and prognosis of tumor patients.

## Author contributions

HW, JW, and CL designed and supervised the review. TL and YL searched the literature and wrote the manuscript. LS and SX searched the literature and reviewed the manuscript. All authors contributed to the article and approved the submitted version.
